# Treatment of Anti-Myelin-Associated Glycoprotein (MAG) Antibody Neuropathy Using Zanubrutinib in a Patient With Waldenström Macroglobulinemia: A Clinical Vignette

**DOI:** 10.7759/cureus.81946

**Published:** 2025-04-09

**Authors:** Romy Pein, Amir Steinberg

**Affiliations:** 1 Hematology and Oncology, New York Medical College, Valhalla, USA; 2 Hematology and Oncology, Westchester Medical Center, Valhalla, USA

**Keywords:** anti-mag neuropathy, bruton’s tyrosine kinase, btk inhibitor, waldenstrom macroglobinaemia, zanubrutinib

## Abstract

The objective of this paper is to report a case of anti-myelin-associated glycoprotein (anti-MAG) antibody neuropathy treated with zanubrutinib, offering insight into a potential therapeutic avenue for this challenging neurological disorder. A 65-year-old male initially presented with peripheral neuropathy in the lower distal extremities. Hematologic evaluation revealed an elevated M-spike of IgM and a high MAG antibody titer. His initial titer was measured at 1:102400 on February 28, 2023. These findings were consistent with the diagnosis of Waldenström macroglobulinemia (WM) with associated anti-MAG antibody neuropathy. The patient was started on rituximab and reported a slight improvement in symptoms. However, the patient felt the efficacy of rituximab diminished with each subsequent dose. Due to this, the patient was initiated on zanubrutinib. Close monitoring of clinical symptoms and laboratory parameters was conducted to assess treatment response and potential side effects. Following zanubrutinib therapy, the patient exhibited mild improvement in neuropathic symptoms, which have stabilized, although still present. Serologic examination demonstrated a decrease in anti-MAG-antibody titer at 1:25600 in the spring of 2023 and 1:51200 in November of 2023. Patient reports increased fatigue and musculoskeletal pain. This clinical vignette highlights the outcomes of zanubrutinib in the management of anti-MAG antibody neuropathy in a patient with WM. Further research and larger clinical trials are warranted to validate these findings and establish zanubrutinib as a viable therapeutic option for this rare and often challenging neurological disorder.

## Introduction

Waldenström macroglobulinemia (WM) is a rare subtype of lymphoplasmacytic lymphoma that results in abnormally high levels of monoclonal IgM antibodies. Approximately 5% of patients with WM will experience neuropathy in reaction to anti-myelin-associated glycoprotein (anti-MAG) antibodies [1]. Studies have estimated that up to 50% of patients with monoclonal gammopathy of undetermined significance (MGUS) may experience an IgM-related neuropathy [2]. Anti-MAG neuropathy is characterized by IgM immunoreactivity to MAG antigens on central and peripheral myelin, leading to paresthesia and discomfort [3]. In more severe cases, the neuropathy can become highly disabling, and previous studies have noted associations between higher age of onset and severity of neuropathy [4]. Anti-MAG neuropathy is often managed with rituximab, a monoclonal antibody that targets CD20 on B cells. However, research has demonstrated that as many as 12% of patients experience worsening of symptoms even while on rituximab [5]. One study examined 10 cases that describe post-rituximab exposure deterioration by means of sensory or motor deficits [6]. Further research suggests that spikes in neuropathic symptoms occur due to IgM flares in patients undergoing rituximab treatment, with a predominant incidence observed in patients with WM [7].

Bruton's tyrosine kinase (BTK) inhibitors have been shown to be a promising treatment option for patients with chronic lymphocytic leukemia (CLL). By selectively inhibiting BTK, a key component of the B-cell receptor signaling pathway, these agents disrupt B-cell activation and proliferation, thereby modulating the immune response. Clinical studies have demonstrated favorable outcomes with BTK inhibitors, such as ibrutinib, in WM patients refractory to or intolerant of standard therapies such as rituximab [8]. In recent years, zanubrutinib, a second-generation BTK inhibitor, has become an emerging therapy for WM. A randomized, phase 3 trial compared zanubrutinib and ibrutinib for patients with WM and found that zanubrutinib demonstrated fewer adverse effects [9]. Zanubrutinib also demonstrated superiority over ibrutinib in patients with refractory CLL [10]. Emerging clinical data indicate that BTK inhibitors show therapeutic potential in the treatment of patients affected by anti-MAG antibody neuropathy, as evidenced by observations within studies of WM patients [11]. There is a class IV evidence study showing that BTK inhibitors can improve anti-MAG-related neuropathy [12]. Here, we describe the clinical course and treatment outcomes of a patient with anti-MAG antibody neuropathy associated with WM, who was prescribed zanubrutinib after experiencing suboptimal response to rituximab.

## Case presentation

A 65-year-old male had a history of hypertension and was a first responder at the World Trade Center attacks on September 11, 2001, and presented with peripheral neuropathy primarily in his feet in November 2019. The symptoms were initially mild but progressed to worsening numbness and tingling with pain in the bilateral feet. The patient reported numbness and tingling in his hands and an occasional tremor. The patient reported that these neuropathic symptoms made it difficult and uncomfortable to go about daily activities. Neurological electromyography (EMG) studies were conducted, which revealed prolonged distal motor latencies and axonal loss. A MAG antibody titer of 1:102400 was detected. He was noted to have a monoclonal spike of 0.8 g/dL, IgM of 390 mg/dL, kappa 2 of 7 mg/L, and a free kappa/lambda ratio of 2.1. A bone marrow biopsy on April 23, 2019, showed IgM-kappa-restricted surface light chain negative low-grade lymphoma, very dim cytoplasmic kappa restricted, CD138 < 5%, CD79a, CD19, CD20 (predominantly strong), CD5 (variable, weak), CD23 (weak), IgM (weak), and kappa was negative for CD3, cyclin D1, lambda, IgG, IgD, IgA, and MYD88 negative. Overall, given the presence of the IgM monoclonal protein and the abnormal B cell population, he was thought to have WM/lymphoplasmacytic lymphoma with associated MAG antibody neuropathy.

The patient was initially treated with rituximab 375 mg/m² weekly for four doses beginning in September of 2020, with subjective response. However, the neurologic symptoms recurred, and he underwent two more courses of four weekly doses of rituximab in July 2021 and September 2021. With each subsequent cycle of rituximab, the patient noted less efficacy in improving his neuropathy. He endorsed continuation of symptoms such as pain in his feet and difficulty buttoning his shirts due to hand stiffness. The patient refused any further EMG testing after the initial study in 2019. After discussion of treatment options, the patient agreed to proceed with zanubrutinib to treat his underlying WM. The hope was that by treating the WM, his neurologic symptoms would improve as well. He was also maintained on duloxetine and pregabalin for pain management, and the doses did not change during this time. He began zanubrutinib in February 2023. Zanubrutinib was prescribed at 320 mg daily orally once a day. The anti-MAG antibody titer at the start of treatment with zanubrutinib was 1:102400 (February 28, 2023). On April 20, 2023, the titer was 1:25600. The level on July 12, 2023, was again 1:25600. On November 7, 2023, the titer was 1:51200. The level on March 7, 2024, was 1:51200. During this time, the patient reported bouts of suboptimal medication adherence. He admitted to cessation of zanubrutinib for several days at a time during August of 2023 and for one week before his visit on November 7, 2023. Through this one-year period, the patient-reported neurologic symptoms had not worsened. The M spike at the time of treatment initiation was 0.2 g/dL. In November 2023, the M protein was 0.1 g/dL. His IgM level also dropped from 811 mg/dL (February 28, 2023) at the initiation of treatment, then 760 mg/dL on April 20, 2023, 738 mg/dL on June 15, 2023, and 712 mg/dL on November 7, 2023. During this time, the patient reported that he felt less shooting pain in his legs when on the zanubrutinib, and the pain in his wrists did not progress the way the leg pain had before (Table 1).

**Table 1:** Patient laboratory values with reference ranges

Laboratory finding	Patient value	Reference value
Anti-MAG antibody titer	1:102400 (February 28, 2023)	<1:160
	1:25600 (April 20, 2023; July 12, 2023)	
	1:51200 (November 7, 2023; March 7, 2024)	
M protein (monoclonal spike)	0.2 g/dL (February 28, 2023)	<0.1 g/dL
	0.1 g/dL (November 7, 2023)	
IgM Level	811 mg/dL (February 28, 2023)	40-230 mg/dL
	760 mg/dL (April 20, 2023)	
	738 mg/dL (June 15, 2023)	
	712 mg/dL (November 7, 2023)	
Free kappa/lambda ratio	2.1	0.26-1.65
Kappa light chain	27 mg/L	3.3-19.4 mg/L

## Discussion

Anti-MAG neuropathy is a debilitating condition that affects about 1 in 100,000 patients suffering from neurological disease. The condition has an estimated mortality rate of 33% at 15 years, highlighting the grim prognosis [13]. Patients with monoclonal IgM proliferation, such as WM, are particularly susceptible to this condition. Several risk factors have been identified for the worsening prognosis of anti-MAG neuropathy, such as a demyelinating pattern and older age. Interestingly, previous studies have shown a lack of significant correlation between the quantity of anti-MAG titer and the severity of disease [4]. Aside from WM, anti-MAG neuropathy has been associated with diseases such as MGUS, CLL, or B-cell lymphoblastic lymphoma (BLL) [14].

Currently, no universally standardized guideline exists for the preferred management of anti-MAG neuropathy in patients with WM [12,15]. Clinical trials examining the effectiveness of rituximab in slowing or reversing the course of neurologic symptoms did not demonstrate significant effectiveness, leaving it to the discretion of the provider to decide if the risks of rituximab outweigh the uncertain benefits [16,17].

As stated previously, BTK inhibitors have become a popular option for treating WM. BTK is a key regulator of several important pathways for B-cell proliferation and differentiation, such as the phosphoinositide 3-phosphate (PIP3) pathway, ras oncogene (RAS), and nuclear factor kappa B (NFkB) [18]. By targeting these mechanisms, BTK inhibitors can reduce the production of IgM antibodies and have shown therapeutic promise in patients [19]. Several studies have examined the responsiveness of rituximab-refractory patients to BTK inhibitors. One of the first-generation BTK inhibitors, ibrutinib, showed significant promise in the iNNOVATE trial that found an 86% progression-free survival rate and 90% response rate in a rituximab-refractory population [20]. Treon et al. demonstrated that in treatment-naïve WM patients with a MYD88 mutation, overall responses were at 100% [21]. MYD88 has an important role in toll-like receptor activation, leading to activation of NFkB. Research has shown that patients with wild-type MYD88 have a more aggressive disease course, resembling that of diffuse large B-cell lymphoma. Furthermore, those patients have been found to have decreased response to ibrutinib [22]. Our patient was MYD88 negative, which was another reason to consider an alternative BTK inhibitor for increased effectiveness.

Ibrutinib, a first-generation BTK inhibitor, has shown promise in improving anti-MAG antibody polyneuropathy. Castellani et al. reported that ibrutinib led to clinical improvement in patients with anti-MAG neuropathy, suggesting its potential to modify the disease course [12]. However, one study found that ibrutinib was tolerated by the patient, but it did not lead to clinical improvement in anti-MAG neuropathy [23]. Tirabrutinib, another BTK inhibitor, has shown promise in treating symptoms related to anti-MAG neuropathy, as reported by Yasuda et al. [19]. These findings indicate that BTK inhibition, in general, may be a beneficial approach for managing this challenging condition.

Direct comparative studies of different BTK inhibitors in anti-MAG neuropathy are limited, likely due to the novelty of this therapeutic approach. Zanubrutinib, a novel Bruton tyrosine kinase (BTK) inhibitor, has garnered considerable attention as a therapeutic agent for WM and anti-MAG neuropathy [24]. While zanubrutinib is being investigated in the MAGNAZ phase II trial for patients with MGUS and anti-MAG neuropathy, the results of this trial are still pending [25].

In a pivotal study led by Dimopoulos et al., zanubrutinib demonstrated remarkable efficacy in patients with relapsed or refractory WM [26]. The trial reported an overall response rate of 81%, with a median 18-month progression-free survival rate of 68%. Notably, zanubrutinib exhibited favorable tolerability, with low rates of treatment discontinuation due to adverse events, emphasizing its potential as a well-tolerated therapeutic option for WM patients. Trotman et al. showed an 80.5% progression-free survival rate in rituximab-refractory patients treated with zanubrutinib [27].

To the best of our knowledge, we believe this to be the first reported use of zanubrutinib to treat anti-MAG neuropathy after refractory responsiveness to rituximab, excluding clinical trials. We identified two previous studies that investigated the role of BTK inhibitors on MAG-antibody titers. One study showed improvement in anti-MAG antibody polyneuropathy with ibrutinib in three patients with anti-MAG neuropathy [12]. A second study from Japan explored the role of tirabrutinib, a second-generation BTK inhibitor, for anti-MAG neuropathy. They reported a 50% reduction in anti-MAG antibody titers in a patient described as having IgM MGUS with anti-MAG neuropathy [19]. There is currently an ongoing study, the MAGNAZ trial, which examines the combination of rituximab and zanubrutinib in treating polyneuropathy associated with anti-MAG antibodies [25]. Due to suboptimal patient adherence and patient reluctance to rituximab from previous experience, the efficacy of a rituximab and zanubrutinib combination regimen could not be evaluated in this case. Our patient initially demonstrated marked improvement after initiation of zanubrutinib treatment by means of varying lower anti-MAG antibody titers. Although his titers increased in late 2023 through 2024, it is important to note that the increase was correlated with patient-reported non-adherence. His symptoms, however, did not worsen over a year of zanubrutinib. The patient endorsed several key symptomatic improvements on zanubrutinib, such as cessation of shooting leg pain and attenuation of hand neuropathy.

## Conclusions

With studies showing the favorable safety of zanubrutinib relative to ibrutinib, the use of zanubrutinib will seem a reasonable option to consider in patients with anti-MAG neuropathy, whether in patients with overt WM or in patients with MGUS with associated neurologic symptoms. Multi-center randomized controlled trials and prospective cohort studies are crucial to thoroughly evaluate the long-term safety profile, including potential rare adverse events, and to confirm sustained efficacy across diverse patient populations. Additionally, research should investigate the optimal integration of this therapy into combination regimens and its potential as an earlier-line treatment strategy, examining its impact on disease progression and long-term patient outcomes.
